# DNA methylation data for identification of epigenetic targets of resveratrol in triple negative breast cancer cells

**DOI:** 10.1016/j.dib.2017.02.006

**Published:** 2017-02-09

**Authors:** Rubiceli Medina-Aguilar, Carlos Pérez-Plasencia, Patricio Gariglio, Laurence A. Marchat, Ali Flores-Pérez, César López-Camarillo, Jaime García Mena

**Affiliations:** a Departmento de Genética y Biología Molecular, Centro de Investigación y de Estudios Avanzados del Instituto Politécnico Nacional (Cinvestav-IPN), México City, México; b Unidad de Biomedicina, FES-Iztacala UNAM, Tlalnepantla, Estado de México, México; c Escuela Nacional de Medicina y Homeopatía. Red de Biotecnología. Instituto Politécnico Nacional, México City, México; d Laboratorio de Oncogenómica y Proteómica del Cáncer, Universidad Autónoma de la Ciudad de México, México City, México

**Keywords:** Breast cancer, Resveratrol, DNA methylation, Oncogenes, Tumor suppressor genes, Genome-wide methylome

## Abstract

Previous studies revealed that some bioactive food components have anti-cancer effects. However epigenetic effects of dietary compound resveratrol are largely unknown in breast cancer cells (M.A. Dawson, T. Kouzarides, 2012) [1]. Here we provide novel data and comparisons of DNA methylation status of promoter gene regions in response to resveratrol treatment at 24 h and 48 h versus untreated MDA-MB-231 breast cancer cells. DNA methylation changes were measured using Array-PRIMES method (aPRIMES) followed by whole-genome hybridization using human DNA methylation promoter microarray NimbleGen HG18 Refseq Promoter 3×720 K array. Our data were associated to corresponding changes in mRNA expression in a set of cancer-related genes. Using gene ontology analysis we also identify cancer-related cellular processes and pathways that can be epigenetically reprogramed by resveratrol. Data in this article are associated to the research articles “Methylation Landscape of Human Breast Cancer Cells in Response to Dietary Compound Resveratrol”. Medina Aguilar et al., PLoS ONE 11(6): e0157866. doi:10.1371/journal.pone.0157866 2016 (A.R. Medina, P.C. Pérez, L.A. Marchat, P. Gariglio, M.J. García, C.S. Rodríguez, G.E. Ruíz, et al., 2016) [2]; and “Resveratrol inhibits cell cycle progression by targeting Aurora kinase A and Polo-like kinase 1 in breast cancer cells” in Oncology Reports. Medina Aguilar et al., 2016 Jun; 35(6):3696-704. doi: 10.3892/or.2016.4728 (A.R. Medina, P. Gariglio, M.J. García, O.E. Arechaga, S.N. Villegas, C.M. Martínez et al., 2016) [3].

**Specifications Table**

**Table:** 

Subject area	Biology
More specific subject area	Breast cancer, Epigenetics
Type of data	Tables, figure, raw data on DNA methylation (peak scores)
How data was acquired	Array-PRIMES method (aPRIMES).
	NimbleGen HG18 Refseq Promoter 3×720 K arrays.
Data format	Filtered for peak annotation, and analyzed for differential enrichment and gene ontology
Experimental factors	DNA from untreated and treated MDA-MB-231 cells with resveratrol (100 µM) at 24 h and 48 h was extracted using the DNeasy Kit (Qiagen, Germany). To identify methylated and unmethylated DNA regions in the promoters of genes, we used Array-PRIMES method (aPRIMES).
Experimental features	MDA-MB-231 cells were treated with resveratrol for 24 h and 48 h. For DNA methylation analysis we used NimbleGen HG18 Refseq Promoter 3×720 K array. The array covering 30,848 transcripts, 22,532 promoters, and 27,728 CpG islands.
Data source location	Oncogenomics and Cancer Proteomics Laboratory, UACM. México City, México.
Data accessibility	Data is available with this article.

**Value of the data**

• First analysis on DNA methylation of promoter genes in triple negative breast cancer cells treated with resveratrol, spanning 27,728 CpG loci.
• Provides genomic data indicating that resveratrol impacts the epigenetic landscape by changing DNA methylation status of specific oncogenes and tumor suppressor genes in breast cancer cells.
• Analyses indicate that resveratrol epigenetically alters regulation of particular genes involved in cancer in triple negative breast cancer cells.
• Data provided here serves as a novel and free resource for researchers working in the field of epigenetic regulation of cancer related genes in response to naturally occurring dietary compounds.

## 1 Data

**Fig. 1:**
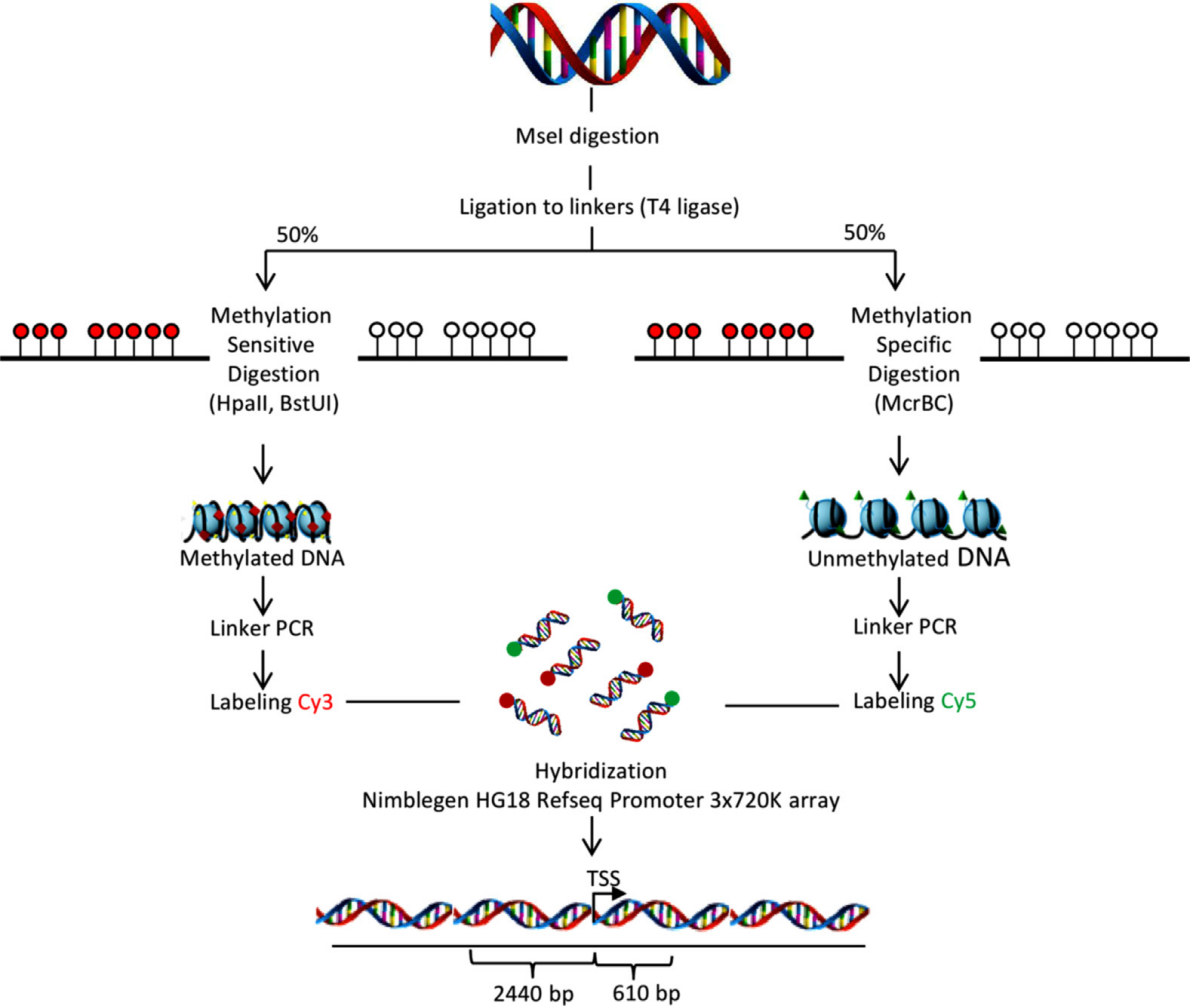
Array-PRIMES procedure and NimbleGen HG18 Refseq Promoter 3×720 K arrays analysis. Genomic DNA (500 ng) was restricted to completion with *Mse*I restriction enzyme. Then, DNA fragments produced by *Mse*I were subjected to linker-mediated PCR. Half of the resulting ligated *Mse*I fragments were digested with *Mcr*BC restriction enzyme while the other half of MseI fragments was digested with the two methylation-sensitive endonucleases *Hpa*II and *Bst*UI. The experimental and control DNA was labeled with Cy3 and Cy5 nonamers. Methylated DNA fractions are highlighted in red and are expected to result in positive ratios and green spots indicate unmethylation and are to have negative ratios. DNA methylation analysis raw data was normalized and differential intensity of each probe compared with input control was calculated using the NimbleGen software DEVA. Average fold change (experimental IP sample versus Control input sample) in a range of 2.44 kb upstream and 610 bp downstream window from RefSeq transcription start sites (TSS). Modified from Pfister, S., Schlaeger, C., Mendrzyk, F., Wittmann, A., Benner, A., Kulozik, A., et al. Array-based profiling of reference-independent methylation status (aPRIMES) identifies frequent promoter methylation and consecutive downregulation of ZIC2 in pediatric medulloblastoma. Nucleic Acids Res. (2007) 35:e51.

In this study we performed a genome-wide survey of DNA methylation in MDA-MB-231 triple-negative breast cancer cells exposed to resveratrol [1], [3]. To determine the methylated and unmethylated DNA regions in the promoters of genes we used Array-PRIMES method (aPRIMES) [[4], Fig. 1]. The extensive annotation of peaks differentially enriched for DNA methylation and peak comparisons between the MDA-MB-231 breast cancer cells treated with resveratrol at 24 h and 48 h versus untreated cells (Table 1, Table 2) were performed using the DEVA 1.2.1 software [2]. At 24 h treatment, 338 out of 2035 hypermethylated genes correspond to cancer genes; and 92 out of 1738 hypomethylated genes correspond to cancer genes. At 48 h treatment, 137 out of 1869 hypermethylated genes were cancer genes; and 288 out of 1661 hypomethylated genes are cancer genes (Table 3, Table 4). In addition, differentially methylated probes and differentially expressed genes were identified between the MDA-MB-231 cells treated with resveratrol at 24 h and 48 h and the MDA-MB-231 untreated. The integrative analysis of DNA methylation and gene expression at different times of resveratrol exposure showed that changes in DNA methylation were associated to corresponding changes in mRNA expression in a set of cancer-related genes (Table 5, Table 6).

## 2 Experimental design, materials and methods

### 2.1 Genome-wide analysis of DNA methylation

High molecular weight DNA from MDA-MB-231 triple negative breast cancer cell line untreated and treated with resveratrol (100 µM) at 24 h and 48 h was extracted using the DNeasyKit (Qiagen, Germany) according to the manufacturer׳s instructions. For detection of the methylation status of CpG islands (CGIs), we used array-based profiling of reference-independent methylation status (aPRIMES) technology in MDA-MB-231 cells untreated and treated with resveratrol at 24 h and 48 h. This method is based on the differential restriction and competitive hybridization of methylated and unmethylated DNA by methylation-specific and methylation-sensitive restriction enzymes, and NimbleGen HG18 Refseq Promoter 3×720 K array to measure the differential DNA methylation as described in Fig. 1. Briefly, genomic DNA (500 ng) was restricted with *Mse*I enzyme (New England Biolabs) and ligated to adapter primers according to the recommendations of the supplier. Then, one-half of the ligated *Mse*I fragments were digested with the methylation-sensitive restriction enzymes *Hpa*II and *Bst*UI to cut unmethylated CGIs, and the remaining half is digested with the methylation-specific enzyme McrBC to cut CGIs methylated. Restricted samples were then subjected to 20 cycles of linker-mediated PCR, differentially labeled with fluorescent dyes Cy3 and Cy5, and competitively hybridized to a NimbleGen HG18 Refseq Promoter 3×720 K array following the conditions recommended by the supplier. DNA methylation analysis raw data was normalized and differential intensity of each probe compared with experimental IP sample (IP) and control input sample (input) was calculated using the NimbleGen software DEVA. Average fold change (IP versus input) each 50 bp bin for a range of 2.44 kb upstream and 610 bp downstream window from RefSeq transcription start sites (TSS). The methylation peak values were mapped to features using DEVA software. Regions showing enrichment at 4 or more consecutive loci were integrated together to form a single “peak”. Clusters of enriched regions separated by more than 500 base pairs were integrated as separate peaks, which reflected the probability of methylation for the designated peak and/or gene at a p-value of less than 0.01. The functional annotation of target genes based on Gene Ontology was performed using DAVID (Database for Annotation, Visualization and Integrated Discovery).

### 2.2 Raw data processing and statistical analysis

A two way ANOVA was performed to identify differentially methylated genes. Only genes with statistically significant differences in DNA methylation levels (*p*-value <0.05) were included.

## Figures and Tables

**Table 1:** Annotation of peak values indicating differentially enriched DNA methylation between the MDA-MB-231 breast cancer cells treated with resveratrol at 24 h versus non-treated cells.

**Chromosome**	**Data start**	**Data end**	**Peak value (≥2)**	**Gene name**	**Accession number**
chr8	1784829	1786208	2,64	ARHGEF10	NM_052838
chrX	1521952	1522785	2,63	ASMTL	NM_004574
chr17	60265001	60266158	2,55	PLEKHM1P	NM_001098813
chr11	125641836	125642888	2,54	SRPR	NM_001113491
chr1	228481793	228482538	2,53	GALNT2	NM_001154458
chr15	98649690	98650344	2,50	ADAMTS17	NM_130786
chr18	74927964	74929029	2,50	ATP9B	NM_033110
chr11	2941097	2942549	2,50	NAP1L4	NM_001080438
chr4	2703310	2703944	2,49	FAM193A	NR_024035
chr17	13340300	13340955	2,49	HS3ST3A1	NM_001086
chr8	6411612	6412359	2,48	MCPH1	NM_014911
chr22	18274404	18275477	2,48	TXNRD2	NM_001088
chr5	1536587	1537240	2,48	LPCAT1	NM_000350
chr7	154718039	154719508	2,46	INSIG1	NM_012089
chr10	120791601	120792250	2,45	EIF3A	NM_203444
chr17	77869583	77871212	2,44	CD7	NM_033151
chr9	115101938	115103549	2,44	RNF183	NM_000392
chr14	104982606	104983241	2,44	MTA1	NM_001023587
chr5	140747519	140748247	2,42	PCDHGB4	NM_001171
chr10	105835961	105837022	2,42	COL17A1	NR_003569
chr8	145642211	145643050	2,41	NFKBIL2	NR_023387
chr4	1798152	1798803	2,41	LETM1	NM_007011
chr19	10113471	10114131	2,41	DNMT1	NM_020676
chr2	121699671	121700316	2,41	TFCP2L1	NM_007313
chr12	119018825	119019566	2,41	RAB35	NM_001003408
chr7	1847487	1848112	2,40	MAD1L1	NM_020469
chr10	90702575	90703440	2,40	ACTA2	NM_001092
chr19	52680045	52681097	2,40	KPTN	NM_032169
chr20	60208995	60209642	2,40	GTPBP5	NM_000017
chr9	129692527	129693188	2,39	ST6GALNAC6	NM_001609
chr10	98730453	98731598	2,39	C10orf12	NM_022735
chr22	17010564	17011897	2,39	USP18	NM_001039844
chr7	128202492	128203241	2,38	OPN1SW	NM_020321
chr17	2264882	2265522	2,38	LOC284009	NM_022914
chr17	61726971	61728024	2,38	PRKCA	NM_000789
chr8	23622819	23623663	2,37	NKX2-6	NM_015831
chr5	43075569	43076724	2,37	C5orf39	NM_147161
chr1	199882046	199882901	2,37	NAV1	NM_018473
chr9	93900029	93900662	2,37	SPTLC1	NM_006821
chr1	24599933	24600693	2,37	C1orf201	NM_007274
chr21	46892750	46893489	2,37	PRMT2	NM_005469
chr3	185026706	185028573	2,36	MAP6D1	NM_001037171
chr2	86104655	86105502	2,36	LOC90784	NM_004035
chr6	3085344	3085995	2,35	BPHL	NM_003501
chr2	65394212	65394839	2,35	SPRED2	NM_001142807
chr1	227471486	227472810	2,34	RAB4A	NM_001111036
chr20	60293922	60294583	2,34	OSBPL2	NM_152282
chr2	27044598	27045242	2,33	MAPRE3	NM_033068
chr3	48454873	48455707	2,33	CCDC51	NM_052957
chr10	1137781	1138492	2,33	WDR37	NM_025149
chr20	56158151	56159807	2,33	C20orf85	NR_023316
chr5	354862	357457	2,32	AHRR	NM_032501
chr9	135267013	135268048	2,32	REXO4	NM_001141945
chr12	103674747	103675398	2,32	CHST11	NM_001101
chr4	38366523	38367250	2,32	KLF3	NM_005159
chr22	18131439	18132080	2,32	TBX1	NM_001615
chr12	1594042	1594714	2,31	WNT5B	NM_016188
chr5	16754086	16754821	2,30	MYO10	NM_006686
chr1	154372213	154372866	2,30	LMNA	NM_001102
chr2	151827549	151828190	2,29	RBM43	NM_024855
chr14	92722258	92722891	2,29	C14orf109	NM_001616
chr7	158405247	158405910	2,29	WDR60	NM_001107
chr8	27907931	27908473	2,29	SCARA5	NM_003474
chr12	131818564	131820443	2,28	ANKLE2	NM_023038
chr6	75968244	75968893	2,28	COL12A1	NM_021794
chr7	143587044	143587802	2,27	OR2A7	NM_139026
chr9	124835446	124837324	2,27	GPR21	NM_139055
chr20	43163505	43164052	2,26	KCNS1	NM_139056
chr17	40925157	40926319	2,26	PLEKHM1	NM_139057
chr7	143578873	143579544	2,26	OR2A20P	NM_021599
chr5	34020027	34021936	2,26	SLC45A2	NM_014694
chr13	112917772	112918407	2,26	CUL4A	NM_006869
chr5	96234968	96236098	2,25	ERAP2	NM_001025107
chr9	90794507	90795655	2,25	C9orf47	NM_001145407
chr14	20225056	20226587	2,25	ANG	NM_018702
chr19	16294245	16294991	2,25	KLF2	NM_052853
chr21	45063979	45064504	2,24	SUMO3	NM_021116
chr16	66500283	66501020	2,24	PSKH1	NM_001116
chr16	12118128	12118835	2,24	SNX29	NM_014190
chr1	200122439	200123474	2,24	SHISA4	NM_144650
chr9	138904005	138904672	2,24	TRAF2	NM_018269
chr2	71544899	71546050	2,24	DYSF	NM_001127687
chr1	163817579	163818316	2,23	LOC400794	NM_024866
chr7	44089644	44090301	2,23	POLM	NM_001081976
chr1	144539510	144540955	2,23	GPR89A	NM_199162
chr6	5080145	5080848	2,23	LYRM4	NM_000679
chr1	2323386	2324029	2,23	RER1	NM_000684
chrX	134257081	134257708	2,23	ZNF75D	NM_001619
chr7	137182964	137184638	2,22	DGKI	NM_007002
chr20	61372176	61373825	2,22	ARFGAP1	NM_001114176
chr3	50351011	50352739	2,22	RASSF1	NM_001134647
chr10	134973176	134974252	2,22	ZNF511	NR_026892
chr3	189377910	189379089	2,22	FLJ42393	NM_152406
chr9	139117469	139118098	2,22	MAN1B1	NM_014423
chr10	12913471	12915172	2,22	LOC283070	NR_003228
chr22	21740231	21741602	2,22	RTDR1	NM_014914
chr16	70115140	70116282	2,22	CHST4	NM_001135189
chr6	132063624	132064382	2,22	OR2A4	NM_018238
chr17	38247695	38249348	2,21	PSME3	NM_001012727
chr5	179209213	179209862	2,21	C5orf45	NM_020132
chr2	218927406	218928519	2,20	C2orf62	NM_020133
chr7	828845	829582	2,20	SUN1	NM_198576
chr1	15955401	15956674	2,20	FBLIM1	NM_001138
chr19	55122520	55123259	2,20	NUP62	NM_000687
chr5	140742359	140743298	2,19	PCDHGA7	NM_006621
chr1	159355730	159356784	2,19	NIT1	NM_020731
chr17	42639212	42640391	2,19	MYL4	NM_001624
chr19	49727224	49727853	2,19	CEACAM20	NM_006303
chr7	4724096	4724845	2,19	FOXK1	NM_001042478
chr17	3652876	3653659	2,19	ITGAE	NM_016282
chr1	145864889	145866055	2,19	GPR89B	NM_003488
chr10	75200738	75201587	2,19	SEC24C	NM_007200
chr10	71601773	71602436	2,19	SAR1A	NM_001136562
chr7	111907572	111908204	2,19	C7orf53	NM_004857
chr10	46501205	46502248	2,19	PPYR1	NM_014371
chr1	154966020	154966748	2,18	C1orf66	NM_001145289
chr5	176870176	176871049	2,18	DDX41	NM_005163
chr12	1574918	1575576	2,18	FBXL14	NM_001098632
chr11	64609997	64610643	2,18	ZFPL1	NM_181690
chr2	99284710	99285565	2,17	LYG1	NM_001012398
chr20	61040935	61041923	2,17	C20orf11	NM_000031
chr18	42165845	42167418	2,17	RNF165	NM_001017423
chr1	31619982	31620649	2,17	FABP3	NM_000693
chr8	141628099	141628779	2,17	EIF2C2	NM_000692
chr5	1687959	1689310	2,17	LOC728613	NM_000691
chr2	96268657	96269891	2,17	LOC285033	NM_001182
chr10	128863488	128864127	2,17	FAM196A	NM_000034
chr4	39158359	39158928	2,17	LOC401127	NM_024105
chr9	135210801	135211972	2,16	SURF1	NM_139178
chr3	113495155	113495827	2,16	SLC9A10	NM_017758
chr17	34137596	34138545	2,16	MLLT6	NM_000697
chr5	72449727	72450772	2,16	TMEM171	NM_001139
chr5	140780698	140781477	2,16	PCDHGA11	NR_002710
chr22	39514927	39515374	2,15	SLC25A17	NM_001140
chr1	225192145	225192870	2,15	CABC1	NM_000698
chr11	65440964	65442337	2,15	C11orf68	NM_001102406
chr7	151790200	151790753	2,15	LOC100128822	NM_020778
chr21	32594464	32595119	2,15	MRAP	NM_139163
chr4	187301973	187302598	2,15	FAM149A	NM_006492
chr14	20930859	20932524	2,14	CHD8	NM_021926
chr2	152664922	152665700	2,14	CACNB4	NM_001633
chr17	7933308	7934143	2,14	ALOX12B	NM_001144
chr1	219121014	219121672	2,14	HLX	NM_030943
chr2	27457537	27459170	2,14	PPM1G	NM_000036
chr17	15463136	15463709	2,14	CDRT1	NM_000480
chr3	73017682	73018439	2,13	GXYLT2	NM_133463
chr22	19383009	19384389	2,13	TMEM191A	NM_001033569
chr19	52711228	52711938	2,13	NAPA	NR_026903
chr13	113157300	113158153	2,13	DCUN1D2	NM_001002244
chr1	16832002	16832449	2,13	CROCCP2	NM_013366
chr10	105606512	105607450	2,13	SH3PXD2A	NM_001145
chr7	6279408	6280147	2,13	CYTH3	NM_015305
chr12	123374687	123375942	2,13	NCOR2	NM_001146
chr16	726949	727598	2,13	NARFL	NM_001118887
chr14	104784128	104785057	2,12	BRF1	NM_001142446
chr21	45185933	45186674	2,12	C21orf70	NM_020987
chr9	99001128	99001684	2,12	ZNF322B	NM_016376
chr11	62403221	62404093	2,12	SLC3A2	NM_054027
chr13	112851148	112851681	2,12	F10	NM_015114
chr20	4648316	4649183	2,12	PRND	NM_016552
chr16	463734	465061	2,12	RAB11FIP3	NM_017664
chr20	48178979	48180250	2,12	TMEM189	NM_013275
chr1	43658960	43660815	2,12	KIAA0467	NM_152345
chr1	90231647	90232482	2,12	GEMIN8P4	NR_026868
chr1	9338507	9339158	2,11	SPSB1	NM_001012421
chr1	201962916	201963647	2,11	ATP2B4	NM_001012419
chr15	61674213	61674782	2,11	FBXL22	NM_001098805
chr9	135214287	135215030	2,11	SURF2	NM_144994
chr11	116606198	116606846	2,11	PCSK7	NM_133475
chr7	23421191	23421918	2,11	IGF2BP3	NR_026556
chr5	180189349	180190218	2,11	LOC729678	NR_027020
chr5	140772911	140773568	2,11	PCDHGA10	NM_153697
chr19	46593106	46593861	2,10	EXOSC5	NM_001115116
chr15	32183123	32183750	2,10	PGBD4	NM_138797
chr7	134112594	134113325	2,10	CALD1	NM_024669
chr15	84102740	84103387	2,10	KLHL25	NM_023016
chr19	2379640	2381804	2,10	LMNB2	NM_001105576
chr4	8504610	8507474	2,10	C4orf23	NM_020140
chr21	31952040	31952696	2,10	SOD1	NM_018043
chr7	100322137	100322774	2,10	SRRT	NM_006305
chr4	14984971	14985856	2,10	C1QTNF7	NM_012403
chr2	27657775	27658608	2,09	C2orf16	NM_012404
chr1	2429943	2430468	2,09	PANK4	NR_003601
chr1	3546593	3547246	2,09	WDR8	NM_174890
chr11	118708974	118709508	2,09	RNF26	NM_005139
chr16	86505816	86506470	2,09	CA5A	NM_001153
chr4	1374349	1375094	2,09	CRIPAK	NM_001040084
chr14	20525685	20527540	2,09	METT11D1	NM_001630
chr3	199363693	199364236	2,09	FAM157A	NM_001637
chr10	133647136	133647878	2,09	BNIP3	NM_001158
chr8	82769155	82769729	2,09	SLC10A5	NR_001557
chr13	32487045	32487700	2,08	KL	NM_001128426
chr19	16001870	16002633	2,08	FLJ25328	NM_012305
chr3	160000866	160001925	2,08	MFSD1	NM_001025205
chr5	55068613	55069836	2,08	DDX4	NM_001077523
chr13	113213013	113213646	2,08	TMCO3	NM_006051
chr8	42413598	42414265	2,08	SLC20A2	NM_005883
chr22	49319516	49320089	2,08	ODF3B	NM_153360
chr9	124682740	124683287	2,08	RC3H2	NM_001640
chr11	74136170	74137222	2,08	RNF169	NM_080649
chr9	128925755	128926851	2,07	RALGPS1	NM_001145646
chr3	150533499	150534068	2,07	TM4SF18	NM_015957
chr12	10972275	10973743	2,07	PRR4	NM_198544
chr2	219816008	219816764	2,07	GLB1L	NM_017413
chr5	99751772	99752407	2,07	LOC100133050	NM_144772
chr6	161502414	161503768	2,07	NCRNA00241	NM_000482
chr17	75803209	75803847	2,07	SGSH	NM_006789
chr17	36558970	36559826	2,07	KRTAP4-5	NM_014508
chr21	32671648	32673291	2,07	URB1	NM_145298
chr20	473097	474051	2,06	CSNK2A1	NM_021822
chr11	128281912	128282465	2,06	KCNJ5	NM_000483
chr17	56835395	56836142	2,06	TBX2	NM_001647
chr17	18371849	18372865	2,06	FAM106A	NM_001638
chr11	63707734	63708189	2,06	STIP1	NM_001136541
chr8	11386701	11387788	2,06	BLK	NM_030643
chr19	7527601	7528351	2,06	PNPLA6	NM_001130415
chr2	69602478	69603137	2,06	AAK1	NM_019101
chr21	44030904	44031743	2,06	RRP1	NM_001136131
chr7	134548861	134549299	2,06	WDR91	NM_018171
chr6	109273995	109274834	2,06	ARMC2	NM_175069
chr11	579622	580285	2,06	PHRF1	NM_001170
chr6	158320472	158322140	2,06	SYNJ2	NR_026558
chr6	160432081	160434242	2,06	LOC729603	NM_001169
chrX	153374208	153374640	2,06	SLC10A3	NM_173800
chr18	13252330	13253283	2,05	C18orf1	NM_001135190
chr12	67014287	67014849	2,05	MDM1	NM_022481
chr22	36858553	36859224	2,05	PLA2G6	NM_018209
chr2	105726392	105727623	2,05	NCK2	NM_006421
chr16	86005066	86005733	2,05	ZCCHC14	NM_012402
chr7	72355435	72356084	2,05	NSUN5	NM_003224
chr11	93939850	93940710	2,05	PIWIL4	NM_000045
chr7	143560250	143560805	2,05	OR2A1	NM_021226
chr7	99534641	99535294	2,04	MCM7	NM_020876
chr10	95712773	95713611	2,04	PIPSL	NM_001007231
chr20	45300989	45301662	2,04	ZMYND8	NM_199282
chr9	99500965	99501616	2,04	XPA	NM_020754
chr9	123698542	123699193	2,04	TTLL11	NM_025251
chr5	153761791	153763648	2,04	GALNT10	NM_181335
chr10	111954922	111956061	2,04	MXI1	NM_014629
chr9	35803160	35804007	2,04	HINT2	NM_018125
chr1	64441661	64442409	2,04	UBE2U	NM_173728
chr20	43949136	43950576	2,04	C20orf165	NM_015320
chrX	48714505	48715134	2,04	GRIPAP1	NM_145735
chr22	18092827	18095050	2,04	GP1BB	NM_006015
chr1	109625594	109626341	2,04	PSRC1	NM_001040025
chr1	68072396	68073070	2,04	GNG12	NM_001037164
chr12	112108623	112109396	2,04	C12orf52	NM_001037174
chr8	144984474	144985327	2,03	PUF60	NM_015161
chr19	45247606	45248839	2,03	ZNF780B	NM_016638
chr9	37775709	37776372	2,03	EXOSC3	NM_032131
chr13	23049798	23050468	2,03	TNFRSF19	NM_025139
chr21	45511110	45511763	2,03	POFUT2	NM_016608
chr5	140752369	140753116	2,03	PCDHGA8	NM_014862
chr12	48964014	48964668	2,03	LIMA1	NM_001178
chr11	116573532	116574183	2,03	SIDT2	NM_152862
chr9	126575311	126575838	2,03	NR6A1	NM_032487
chr6	18494407	18494835	2,03	RNF144B	NM_001080523
chr22	19717794	19718568	2,03	SLC7A4	NM_001085427
chr2	190979585	190980238	2,03	MFSD6	NM_000047
chr8	23484771	23485496	2,03	SLC25A37	NM_001012301
chr6	1550629	1552574	2,03	FOXC1	NM_021071
chr8	146021440	146021889	2,03	ZNF7	NM_139058
chr7	72064829	72065286	2,03	NSUN5P2	NM_018482
chr9	129582203	129583046	2,02	SH2D3C	NM_001135191
chr10	74926545	74927317	2,02	USP54	NM_001040445
chr1	226361765	226362490	2,02	MRPL55	NM_024701
chr7	98470784	98471533	2,02	SMURF1	NM_212556
chr7	100675731	100676278	2,02	FIS1	NM_016150
chr3	129709277	129710016	2,02	LOC90246	NM_016116
chr18	69967812	69968570	2,02	C18orf55	NM_177999
chr19	2750403	2751132	2,02	THOP1	NM_014034
chr4	7804401	7805550	2,02	AFAP1AS	NM_004674
chr20	51926997	51927566	2,02	SUMO1P1	NM_001672
chr3	197777980	197778910	2,02	WDR53	NM_004043
chr11	66245244	66246291	2,02	SPTBN2	NM_004192
chr19	2082237	2082992	2,02	AP3D1	NM_152792
chr11	71492967	71494231	2,02	LRTOMT	NM_024083
chr5	114628333	114628885	2,01	PGGT1B	NM_014065
chr6	90081076	90081725	2,01	GABRR2	NM_198186
chr7	149731981	149732428	2,01	LOC728743	NM_018188
chr22	37738715	37739318	2,01	APOBEC3C	NM_031921
chr20	37022165	37022900	2,01	DHX35	NM_001039211
chr11	57262894	57263532	2,01	TMX2	NM_033064
chr13	113143850	113144500	2,01	ADPRHL1	NM_001030287
chr17	4554829	4555791	2,01	PELP1	NM_030803
chr8	103946748	103947289	2,01	AZIN1	NM_033388
chr2	70635358	70636107	2,01	TGFA	NM_024085
chr19	16145082	16145738	2,01	CIB3	NM_024490
chr9	115702649	115703348	2,01	ZNF618	NM_032189
chr6	33278127	33278983	2,01	SLC39A7	NM_024524
chr6	44228307	44228832	2,01	TMEM63B	NM_000702
chr10	35574617	35575279	2,01	CCNY	NM_001135765
chr11	130292597	130293452	2,01	SNX19	NM_174953
chr17	36507252	36508001	2,01	KRTAP4-8	NM_001001396
chr5	111123614	111124276	2,01	C5orf13	NM_001002031
chr11	124050683	124051236	2,00	SPA17	NM_001003713
chr4	6975976	6976625	2,00	TBC1D14	NM_001017971
chr14	19994926	19995487	2,00	APEX1	NM_005765
chr2	68813855	68814386	2,00	ARHGAP25	NM_012463
chr10	106084059	106084715	2,00	ITPRIP	NM_152565
chr1	59532773	59533669	2,00	FGGY	NM_145230
chr10	126701787	126702422	2,00	CTBP2	NM_001695
chr7	149655954	149657392	2,00	LRRC61	NM_144583
chr1	9593002	9593729	2,00	TMEM201	NM_080653
chr17	75364548	75365507	2,00	CBX2	NM_138282
chr1	40191655	40192402	2,00	MFSD2A	NM_015941
chr4	39721670	39722341	2,00	LOC344967	NM_001105529
chr1	25106856	25107403	2,00	RUNX3	NM_138813

**Table 2:** Annotation of peak values indicating differentially enriched DNA methylation between the MDA-MB-231 breast cancer cells treated with resveratrol at 48 h versus non-treated cells.

**Chromosome**	**Data start**	**Data end**	**Peak value (≥ 2)**	**Gene name**	**Accession number**
chr20	51631293	51631942	2,33	ZNF217	NM_006526
chr7	129718140	129719263	2,33	CPA4	NM_016352
chr14	104311019	104311666	2,32	AKT1	NM_005163
chr1	148511761	148512420	2,31	C1orf54	NM_024579
chr14	23710633	23711373	2,31	REC8	NM_005132
chr2	151827549	151828190	2,30	RBM43	NM_198557
chr7	158509814	158510485	2,27	LOC154822	NR_024394
chr17	23822375	23823405	2,26	SLC13A2	NM_001145976
chr14	95011537	95012580	2,24	C14orf49	NM_152592
chr14	22458926	22460289	2,24	PRMT5	NM_006109
chr10	99197678	99198211	2,24	ZDHHC16	NM_198046
chr2	238430809	238431646	2,20	RAMP1	NM_005855
chr1	43198303	43199496	2,20	SLC2A1	NM_006516
chr6	143931574	143932238	2,19	LOC285740	NR_027113
chr8	131022998	131023436	2,17	FAM49B	NM_016623
chr8	22024534	22025287	2,17	NUDT18	NM_024815
chr11	63747908	63749057	2,16	TRPT1	NM_031472
chr4	3496220	3496869	2,16	LRPAP1	NM_002337
chr7	34840781	34841438	2,15	NPSR1	NM_207173
chr5	94752293	94753044	2,15	FAM81B	NM_152548
chr1	199882134	199882991	2,14	NAV1	NM_020443
chr1	207865542	207866280	2,14	LAMB3	NM_001017402
chr16	16231473	16232198	2,14	NOMO3	NM_001004067
chr1	66230912	66231596	2,13	PDE4B	NM_001037341
chr8	86478784	86479521	2,13	CA1	NM_001128829
chr2	11210550	11211221	2,13	PQLC3	NM_152391
chr1	11843395	11843983	2,12	NPPB	NM_002521
chr20	36486806	36487535	2,12	SNORA71B	NR_002910
chr3	39125418	39126567	2,12	TTC21A	NM_145755
chrX	99080841	99081873	2,12	LOC442459	NR_024608
chr20	51926997	51927566	2,11	SUMO1P1	NR_002189
chr6	167718803	167720348	2,11	TCP10	NM_004610
chr7	75207943	75208630	2,10	HIP1	NM_005338
chr3	113495155	113495728	2,10	SLC9A10	NM_183061
chr17	8811092	8811749	2,10	PIK3R5	NM_001142633
chrX	69199270	69200037	2,10	OTUD6A	NM_207320
chr14	90790629	90792432	2,09	GPR68	NM_003485
chr19	14042256	14043117	2,09	LOC113230	NR_024282
chr1	177825607	177826556	2,09	TDRD5	NM_173533
chr11	73337521	73338348	2,08	DNAJB13	NM_153614
chr13	77212180	77213337	2,08	SLAIN1	NM_001040153
chr2	74607544	74609104	2,07	AUP1	NM_181575
chr16	30301072	30301723	2,06	Septin 1	NM_052838
chr6	3085344	3085995	2,06	BPHL	NR_026650
chr20	36506395	36506925	2,06	SNHG11	NR_003239
chr19	53768383	53768955	2,06	SULT2B1	NM_177973
chr20	3945117	3945570	2,05	RNF24	NM_001134338
chr1	199129316	199130071	2,05	C1orf106	NM_018265
chr17	36276397	36277040	2,05	KRT12	NM_000223
chr5	6500743	6501588	2,04	UBE2QL1	NM_001145161
chr2	74533281	74533854	2,02	INO80B	NM_031288
chr7	100092951	100093994	2,02	ACTL6B	NM_016188
chr1	109835741	109836594	2,02	ATXN7L2	NM_153340
chr10	127455757	127456800	2,02	MMP21	NM_147191
chr11	19327809	19328980	2,02	NAV2	NM_001111018
chr11	62147480	62147867	2,02	B3GAT3	NM_012200
chr2	127892455	127893118	2,02	PROC	NM_000312
chr20	17622453	17622899	2,01	BANF2	NM_001159495
chr11	69934732	69935465	2,01	CTTN	NM_005231
chr15	70278412	70279371	2,01	PKM2	NM_182470
chr12	48782280	48783245	2,01	GPD1	NM_005276
chr1	150753273	150754018	2,01	CRCT1	NM_019060
chr1	54786266	54787121	2,01	ACOT11	NM_147161
chr14	19994926	19995487	2,00	APEX1	NM_080649
chr1	36320120	36321620	2,00	TEKT2	NM_014466

**Table 3:** List of hypermethylated and hypomethylated cancer genes in MDA-MB-231 cells expose to resveratrol at 24 h.

Out of 2035 Hypermethylated genes, 338 were cancer-related genes:	ABCC5, ABL1, ACD, ACE, ACTB, ACTL7B, ACVR2A, ADCY1, AFF4, AJAP1, AKAP13, AKT1, ALOX12, ANGPT1, ANK3, ANKLE2, ANKRD24, APOBEC2, ARID1A, ARNT2, ASPSCR1, ATP10A, ATP2A2, ATXN3L, AXIN1, BCL2L10, BCL7A, BCOR, BMPR1A, BRD3, BTG2, C10ORF119 (MCMBP), C10ORF2, C21ORF29 (TSPEAR), C3, CACNA1D, CACNA1H, CAMTA1, CARD11, CARS, CBFA2T3, CBLB, CCDC41 (CEP83), CCDC6, CCDC81, CCNL1, CCR5, CDC25C, CDC42BPB, CDC73, CDHR2, CDHR3, CDK6, CDKN1A, CDYL, CHD3, CHD4, CIC, CNTNAP1, COL1A1, COL4A2, COL5A1, CREB3L1, CREBBP, CRLF2, CRTC1, CSDE1, CSH1, CSNK2B, CTCF, CYFIP1, CYLD, CYTSB (SPECC1), DCAF4L2, DCHS1, DCTN1, DDX41, DICER1, DIP2C, DLC1, DLEU2, DLG3, DNMT1, DNMT3A, DOCK8, DOT1L, DPP6, DRD5, DST, DYNC1I1, EGFR, EIF1AX, EIF3A, ELN, EPB41L3, EPHA1, EPHB4, ESPL1, EXOC2, EYA4, EYS, EZH2, F8, FAM123B (AMER1), FAM171A1, FAM22A (NUTM2A), FAM46C, FAT4, FBXO32, FGF19, FGFR2, FGFR3, FGFR4, FLCN, FMN2, FOXO1, FRMD7, FYN, FZD3, GATA2, GLI3, GLIPR2, GLTSCR1, GML, GNA11, GPR123, GRB2, HDAC9, HERC2, HIP1, HIST1H2AL, HIST1H3D, HLF, HOXA7, HOXD13, HPD, HSP90AA1, IDH2, IL1B, IRF2, ITGA4, JAK2, KBTBD11, KCNJ5, KDM2A, KDM2B, KDM5C, KDSR, KIAA0427 (CTIF), KIAA1024, KIAA1549, KIAA1751 (CFAP74), KIAA1804, KIF1B, KIF5B, KLF2, KLF6, KLHDC4, KLHL6, KNDC1, LDB1, LETM1, LRP2, LTK, MACF1, MAFA, MAP2K2, MAP3K1, MED1, MEN1, MLL (KMT2A), MLL3 (KMT2C), MLLT1, MLLT4, MLLT6, MMP13, MN1, MNX1, MPL, MRPS31, MSH2, MSN, MTOR, MTUS2, MYH11, MYO18B, MYO1B, MYO1G, MYO9A, MYST3 (KAT6A), NCOR2, NEURL4, NF2, NFE2L3, NIN, NLRP3, NOTCH1, NOTCH2, NOTCH3, NR4A3, NSD1, NTRK1, NUAK1, NUDT14, NUP98, ODZ2 (TENM2), OLIG2, OR2A42, OTOP1, PABPC1, PAG1, PAIP1, PAX5, PAX8, PBX1, PCSK6, PCSK7, PDCD6, PDE4DIP, PDGFB, PDGFRA, PDGFRB, PDZD2, PFKP, PHC2, PI4KA, PIK3AP1, PIK3C2B, PIK3CD, PIK3CG, PIK3R1, PKD1L2, PKP4, PLB1, PLCG1, PLXNA2, PLXNA3, PLXND1, PMS2, PNKD, PNN, POU5F1, PPARG, PPM1D, PRDM16, PRIC285 (HELZ2), PRKACA, PRKAR1A, PRKX, PTPN14, PTPRB, PTPRD, PXDN, RABEP1, RADIL, RALGDS, RBMX, RECQL4, RET, RGS12, RHOB, RHOH, RIPK1, RNF213, RNF43, RPS27, RPTOR, RUFY1, RUNDC2A (SNX29), RUNX1, RXRA, RYR2, SCN11A, SCN5A, SDHAF2, SDHB, SEPT7P2, SGK1, SH2B3, SH3GL1, SLC12A6, SLC9A3R1, SMARCA4, SMARCE1, SMC3, SORCS2, SOX2, SRCAP, SRSF3, SS18L1, ST6GAL2, STAT3, STC1, STK19, STYK1, SYNE2, TAF1, TAL1, TAL2, TBX18, TCHH, TERT, TFDP1, TFPT, TLL2, TLX3, TMEM132D, TMSL3 (TMSB4XP8), TNFAIP3, TNFRSF17, TNFSF8, TOP1, TP53, TPM4, TRAF7, TRAK1, TRRAP, TTC18 (CFAP70), TUBA3C, U2AF1, UBR5, VWF, WAC, WAS, WDFY3, WHSC1, WIPF2, WNT2, WT1, XBP1, XPA, XPC, ZFR2, ZFX, ZNF331, ZNF469, ZNF497, ZNF750.
Out of 1738 Hypomethylated genes, 92 were cancer-related genes:	ABCA7, ABLIM2, AHNAK, ALS2CL, ARHGAP28, ATP2B3, ATP2C2, ATRX, C19ORF26, C1QL2, C2CD4C, CCDC63, CCND1, CCT8L2, CD79B, CDK13, CHD7, CHST1, CLK3, CNGA4, COL9A2, COX6C, CRTC3, CSMD3, CTNNA2, CYP2E1, DCAF12L2, DDB2, EGFLAM, ERCC2, ERCC6, ETV5, EVPL, FAM58A, FLI1, FLT1, FOXO3, FTCD, GNAT1, HCN1, HOXA9, IGF2, IGF2R, IKZF1, IRF4, KEAP1, KHSRP, LIFR, LMX1A, LPHN1, MED12, MSR1, MST1, MST1R, MUC6, MYCL1 (MYCL), MYD88, MYH9, MYOM2, NEUROG2, NRG2, OR51I2, P2RY8, PCDHB6, PHOX2B, PLCH2, POLE, PPP1R3A, PPP6R2, PROM1, RASGEF1A, RFTN1, RPL5, RYR1, SALL3, SEMA5B, SETBP1, SIGLEC1, SOX9, SPTBN4, STK11, TCF3, TLR2, TMPRSS2, TNFRSF14, TRAF5, TSC2, WDR24, WIF1, ZMYND10, ZNF536, ZZEF1.

**Table 4:** List of hypermethylated and hypomethylated genes in MDA-MB-231 cells expose to resveratrol at 48 h.

Out of 1869 Hypermethylated genes, 137 were cancer-related genes:	ABCC5, ACSL6, AFF4, AKAP8, AKT1, ARHGAP32, ARID1B, ATP4A, BCL10, BCL11B, BPTF, BUB1B, C14ORF49 (SYNE3), CACNA1D, CALCR, CANT1, CARD10, CCDC41 (CEP83), CCNB1IP1, CDHR2, CLP1, COL1A1, CSF3R, CSH1, CSMD1, CXCR7 (ACKR3), DLG3, DNMT3A, DOCK2, DOCK8, DSE, EDNRB, EGR2, EIF1AX, EPHA1, ERG, EZH2, FAM171A1, FAM92B, FANCA, FGFR1, FMN2, G6PC, GLI3, GNA11, GNA13, GRB2, GRM8, HEATR7B2 (MROH2B), HERPUD1, HIP1, HIST1H2AL, HIST1H2BG, HIST1H4D, HOXA13, HOXD13, IRX6, ITGA4, ITGB1BP3 (NMRK2), JAK3, KALRN, KIAA1549, KIAA1804, KLHL6, LAMA4, LCK, MAFB, MAP2K3, MAP7, MCHR1, MED1, MED12L, MEF2C, MSH2, MTOR, MTUS2, MUC2, MYH11, MYO18B, MYO1B, NEK2, NEURL4, NFE2L2, NTRK1, NUAK1, NUP98, ODF4, OFD1, PABPC1, PAX8, PCDH18, PDCD6, PDHB, PIK3R5, PKD1L2, PLB1, PMS2, PNN, PNPLA7, PRF1, PROKR2, PTEN, PTPN12, RAC1, RALGDS, RET, RIPK1, RNF103, RUFY1, RUNX1, SATB2, SDHB, SDHD, SHANK1, SLC25A48, SMARCA4, SSX1, STAT6, SYK, TET3, TGFBR2, TLX3, TNFRSF17, TNFSF8, TPM3, TRAK1, TRIM33, TRPS1, TRPV6, U2AF1, U2AF2, UBE2A, UBE2D1, VANGL1, VTI1A, ZFX, ZNF765.
Out of 1661 Hypomethylated genes, 288 were cancer-related genes:	ABCA7, ABL1, ABLIM2, ACD, AHNAK, AJAP1, ALOX12, ALS2CL, ANGPT1, ANK3, ANKLE2, ANKRD24, APOBEC2, ARHGAP28, ASPSCR1, ATP10A, ATP2A2, ATP2B3, ATP2C2, ATRX, AXIN1, BCL2L10, BCL7A, BCOR, BRD3, BTG2, C10ORF2, C19ORF26, C1QL2, C21ORF29 (TSPEAR), C2CD4C, C3, CACNA1H, CARS, CBFA2T3, CCDC6, CCDC63, CCDC81, CCND1, CCNL1, CCT8L2, CD79B, CDC42BPB, CDC73, CDK13, CDKN1A, CDYL, CHD3, CHD4, CHD7, CHST1, CLK3, CNGA4, CNTNAP1, COL4A2, COL5A1, COL9A2, COX6C, CREBBP, CRLF2, CRTC1, CRTC3, CSMD3, CSNK2B, CTNNA2, CYFIP1, CYP2E1, CYTSB (SPECC1), DCAF12L2, DCHS1, DCTN1, DDB2, DDX41, DICER1, DIP2C, DLC1, DLEU2, DNMT1, DOT1L, DPP6, DRD5, DST, EGFLAM, EGFR, EIF3A, ELN, EPHB4, ERCC2, ERCC6, ESPL1, ETV5, EVPL, EXOC2, EYS, F8, FAM22A (NUTM2A), FAM46C, FAM58A, FAT4, FGFR2, FGFR3, FGFR4, FLCN, FLI1, FLT1, FOXO1, FOXO3, FRMD7, FTCD, FYN, GATA2, GLIPR2, GML, GNAT1, GPR123, HCN1, HDAC9, HERC2, HIST1H3D, HLF, HOXA7, HOXA9, HPD, HSP90AA1, IDH2, IGF2, IGF2R, IKZF1, IL1B, IRF2, IRF4, JAK2, KCNJ5, KDM2B, KDM5C, KDSR, KEAP1, KHSRP, KIAA0427 (CTIF), KIAA1751 (CFAP74), KIF1B, KLF2, KLHDC4, KNDC1, LDB1, LETM1, LIFR, LMX1A, LPHN1, LTK, MAFA, MAP2K2, MED12, MEN1, MLL (KMT2A), MLLT1, MLLT6, MMP13, MNX1, MSN, MSR1, MST1, MST1R, MUC6, MYCL1 (MYCL), MYD88, MYH9, MYO1G, MYOM2, NCOR2, NEUROG2, NF2, NIN, NLRP3, NOTCH1, NOTCH3, NR4A3, NRG2, NSD1, NUDT14, ODZ2 (TENM2), OLIG2, OR2A42, OR51I2, P2RY8, PAG1, PAX5, PBX1, PCDHB6, PCSK6, PCSK7, PDE4DIP, PDGFRA, PDGFRB, PDZD2, PFKP, PHC2, PHOX2B, PI4KA, PIK3AP1, PIK3CG, PIK3R1, PLCG1, PLCH2, POLE, POU5F1, PPARG, PPM1D, PPP1R3A, PPP6R2, PRDM16, PRIC285 (HELZ2), PRKX, PROM1, PTPN14, PTPRB, PTPRD, PXDN, RADIL, RASGEF1A, RBMX, RFTN1, RGS12, RHOB, RHOH, RNF213, RNF43, RPL5, RPTOR, RUNDC2A (SNX29), RXRA, RYR1, SALL3, SCN11A, SCN5A, SDHAF2, SEMA5B, SEPT7P2, SETBP1, SH3GL1, SIGLEC1, SMC3, SORCS2, SOX9, SPTBN4, SRCAP, SS18L1, ST6GAL2, STC1, STK11, STK19, SYNE2, TAL2, TBX18, TCF3, TCHH, TERT, TFDP1, TFPT, TLL2, TLR2, TMEM132D, TMPRSS2, TMSL3 (TMSB4XP8), TNFRSF14, TOP1, TP53, TRAF5, TRAF7, TRRAP, TSC2, TTC18 (CFAP70), TUBA3C, WAS, WDR24, WHSC1, WIF1, WIPF2, WT1, XPA, XPC, ZFR2, ZMYND10, ZNF331, ZNF469, ZNF497, ZNF536, ZZEF1.

**Table 5:** Integrative analysis of DNA methylation and gene expression at 24 h of resveratrol exposure.

**Gene name**	**Fold change**	**Peak value**	**Gen expression/ Methylation status**
AURKA	−1,86	1,24	Low/hypermethylated
CCNB1	−1,54	1,56	Low/hypermethylated
DDIT4	−2,82	1,8	Low/hypermethylated
DLGAP5	−1,57	1,63	Low/hypermethylated
EYS	−1,62	1,59	Low/hypermethylated
FAM83D	−1,52	1,33	Low/hypermethylated
HIST1H2BK	−1,52	1,62	Low/hypermethylated
HIST1H2BM	−6,63	1,29	Low/hypermethylated
IL24	−2,84	1,49	Low/hypermethylated
LPXN	−1,52	1,88	Low/hypermethylated
NFIL3	−1,5	1,47	Low/hypermethylated
PFKFB3	−1,65	1,5	Low/hypermethylated
SLC14A1	−1,68	1,24	Low/hypermethylated
STC1	−1,69	1,2	Low/hypermethylated
AMY1A	1,56	−1,31	High/hypomethylated
IL18	1,55	−1,12	High/hypomethylated
PEG10	1,61	−1,42	High/hypomethylated
SLIT3	2,13	−1,34	High/hypomethylated
TTI1	1,59	−1,65	High/hypomethylated
WDR52	1,65	−1,17	High/hypomethylated

**Table 6:** Integrative analysis of DNA methylation and gene expression at 48 h of resveratrol exposure.

**Gene name**	**Fold change**	**Peak**	**Gen expression/ Methylation status**
GPR110	−2,25	1,1063	Low/hypermethylated
HIST1H3F	−1,65	1,5632	Low/hypermethylated
HK2	−2,03	1,0942	Low/hypermethylated
MMP9	−1,61	1,7961	Low/hypermethylated
NEDD4	−1,5	1,1856	Low/hypermethylated
NFIL3	−1,5	1,3398	Low/hypermethylated
PSMD11	−1,54	1,5088	Low/hypermethylated
RUNX2	−1,54	1,3272	Low/hypermethylated
SH3KBP1	−1,55	1,487	Low/hypermethylated
ANKRD20A3	2,14	−0,1	High/hypomethylated
MPHOSPH9	1,56	−0,39	High/hypomethylated
PEG10	2,1	−0,9	High/hypomethylated
SLC27A2	1,56	−0,26	High/hypomethylated
SLIT3	1,88	−0,12	High/hypomethylated
TMOD2	1,56	−0,19	High/hypomethylated
TTI1	1,75	−0,27	High/hypomethylated
XYLB	1,51	−0,35	High/hypomethylated
