# Supplementary material for: DNA methylation data for identification of epigenetic targets of resveratrol in triple negative breast cancer cells
Source: Data Brief. 2017 Feb 9;11:169–82. doi: 10.1016/j.dib.2017.02.006 (PMC5312642; doi:10.1016/j.dib.2017.02.006)
Supplement: Supplementary file 1 — Supplementary material [file mmc1.docx]

**DNA methylation data for identification of epigenetic targets of resveratrol**

**in triple negative breast cancer cells**

**Authors:**

Rubiceli Medina-Aguilar^1^, Carlos Pérez-Plasencia^2^, Patricio Gariglio^1^, Laurence A. Marchat^3^, Ali Flores-Pérez^4^, César López-Camarillo^4^*, Jaime García Mena^1^**

**Conflicts of Interest Statement**

The authors who have taken part in this study declared that they do not have anything to disclose regarding funding from industry or conflict of interest with respect to this manuscript.
